# Editorial: The Application of EEG in Neurorehabilitation

**DOI:** 10.3390/brainsci15080856

**Published:** 2025-08-12

**Authors:** Ryouhei Ishii

**Affiliations:** 1 Department of Occupational Therapy, Osaka Metropolitan University Graduate School of Rehabilitation Science, 3-7-30 Habikino, Habikino 583-0872, Osaka, Japan; ishii@psy.med.osaka-u.ac.jp; Tel.: +81-72-950-2111; Fax: +81-72-950-2131; 2 Department of Rehabilitation, Osaka Kawasaki Rehabilitation University, 158 Mizuma, Kaizuka 597-0104, Osaka, Japan; 3 Department of Psychiatry, Osaka University Graduate School of Medicine, 2-2 Yamadaoka, Suita 565-0871, Osaka, Japan

## 1. Introduction

The field of neurorehabilitation stands at a pivotal juncture, driven by a confluence of advancements in neuroscience, engineering, and data science. At the heart of this transformation is the imperative to move beyond one-size-fits-all therapeutic models towards personalized, evidence-based interventions that are precisely tailored to the individual’s unique neural landscape. Electroencephalography (EEG), a technology with a long and storied history in clinical neurology, has re-emerged as a powerful and versatile tool in this new era. Its non-invasive nature, exceptional temporal resolution, portability, and relative affordability have positioned it as an ideal modality for exploring the dynamic processes of neural recovery and guiding rehabilitation strategies. This Special Issue of *Brain Sciences*, titled “The Application of EEG in Neurorehabilitation”, brings together a collection of cutting-edge research that collectively illuminates the expanding role of EEG in assessing brain function, optimizing therapeutic interventions, and pioneering the next generation of assistive technologies [1].

For decades, the primary application of EEG in neurology was diagnostic, focused on identifying epileptiform activity or characterizing sleep stages. However, the last two decades have witnessed a paradigm shift. The integration of sophisticated signal processing algorithms, machine learning, and mobile brain/body imaging (MoBI) has unlocked the potential of EEG to serve not just as a diagnostic tool but as a dynamic window into the functioning brain [2]. This is particularly crucial in neurorehabilitation, where the ultimate goal is to understand and promote neural plasticity—the brain’s intrinsic ability to reorganize itself following injury from events such as stroke, traumatic brain injury (TBI), or in the context of neurodevelopmental conditions such as cerebral palsy [3].

Despite this progress, significant gaps in our knowledge and technical challenges remain. One of the most persistent hurdles is inter-subject variability; neural signatures that are robust in one individual may be weak or absent in another, complicating the development of universally effective brain–computer interfaces (BCIs) or biomarkers [4]. Furthermore, translating findings from controlled laboratory settings into complex, real-world clinical environments remains a formidable challenge [5]. There is a pressing need for more robust, interpretable models that can handle the noisy, high-dimensional data characteristic of EEG recordings during movement and interaction. Additionally, while we have become proficient at identifying neural correlates of motor function, the neurophysiological underpinnings of cognitive and motivational factors—which are critical for patient engagement and successful rehabilitation—are less understood.

This Special Issue directly confronts these challenges, presenting a multifaceted view of how researchers are pushing the boundaries of what is possible with EEG. The nine articles featured herein span a wide spectrum of applications, from foundational investigations into neural mechanisms to the development of advanced computational models and the exploration of novel therapeutic paradigms.

## 2. Advancing Brain–Computer Interfaces and Computational Models

A central theme of this collection is the relentless pursuit of more accurate and reliable BCI systems, which hold immense promise for restoring function to individuals with severe motor impairments. Three papers in this Special Issue tackle key challenges in BCI development from a computational perspective. Mao et al. (Contribution 1) address the critical issue of subject-independent EEG recognition in motor imagery tasks. Their proposed Mirror Contrastive Learning with Sliding Window Transformer (MCL-SWT) model is a significant step forward, leveraging neurophysiological principles (contralateral event-related desynchronization) to inform the architecture of their deep learning model. By improving performance on benchmark datasets, their work paves the way for BCIs that require less user-specific calibration, a major barrier to clinical adoption.

Complementing this, Suresh et al. (Contribution 2) explore the synergy between EEG-based machine learning and non-invasive brain stimulation (NIBS). Their study demonstrates that transcranial direct current stimulation (tDCS) not only facilitates motor recovery but also enhances the decodability of movement-related EEG signals in chronic stroke patients. This finding is profound, suggesting that NIBS can be used to “prime” the brain, making neural states more distinct and therefore easier to classify. This creates a potential positive feedback loop where stimulation improves both neural function and the BCI’s ability to assist that function. The work by Adolf et al. (Contribution 3) further underscores the complexity of the BCI pipeline, conducting a systematic investigation into how different preprocessing choices—such as artifact rejection, filtering, and transfer learning—impact classification accuracy. Their results highlight that there is no single “best” pipeline; rather, processing techniques must be carefully selected and tailored to specific network architectures and even individual subjects, reinforcing the need for personalized approaches.

## 3. Uncovering the Neural Signatures of Motor Control and Recovery

To build effective rehabilitation strategies, we must first understand the fundamental neural mechanisms of motor control and how they are altered by injury. Okuyama et al. (Contribution 4) provide a blueprint for this by investigating the cortical networks underlying stepping accuracy in healthy individuals using mobile EEG. Their work, integrating eLORETA-ICA with microstate analysis, reveals a distributed network where the anterior cingulate cortex (ACC) plays a central role in performance monitoring. This provides a normative model against which patient populations can be compared, offering potential targets for therapeutic intervention aimed at improving gait and preventing falls.

Lacerda et al. (Contribution 5) apply a similar neurophysiological lens directly to stroke survivors. Their longitudinal study powerfully demonstrates the clinical utility of EEG- and TMS-derived biomarkers. They identify the theta/alpha ratio (TAR) in the lesioned hemisphere as a robust predictor of motor outcomes and a potential signature of adaptive compensatory processes. This research exemplifies the goal of translational neuroscience: to identify reliable biomarkers that can predict recovery trajectories and help clinicians stratify patients for targeted therapies, moving us closer to a precision medicine model for stroke rehabilitation.

## 4. Exploring the Psycho-Social and Contextual Dimensions of Rehabilitation

Successful rehabilitation is not merely a matter of retraining motor circuits; it is deeply influenced by motivation, social interaction, and cognitive engagement. Two particularly innovative articles in this Special Issue use EEG to explore this often-overlooked dimension. Yamauchi et al. (Contribution 6) investigate how the brains of children with cerebral palsy respond differently to verbal encouragement from their mothers versus their physical therapists. Their findings suggest that the source and tone of voice elicit distinct patterns of neural activity, with a mother’s voice potentially engaging more internal, self-referential processing. This work highlights the critical importance of the therapeutic alliance and communication in pediatric rehabilitation.

Similarly, Ozel (Contribution 7) uses a virtual reality (VR) environment to dissect how the brain processes social versus non-social cues during a working memory task. By creatively applying Leading Eigenvector Dynamics Analysis (LEiDA) to EEG data, the study reveals that social avatar cues trigger a unique brain state characterized by heightened connectivity in self-referential and memory networks. This suggests that embedding social elements into VR-based rehabilitation tasks could significantly enhance cognitive engagement and, by extension, therapeutic outcomes.

## 5. Foundational Research for Future Therapies

Finally, this Special Issue includes two contributions that lay the groundwork for future therapeutic development. Morales Fajardo et al. (Contribution 8) conduct a focused investigation into the effects of high-definition transcranial alternating current stimulation (HD-tACS) on beta oscillations in healthy older adults. By demonstrating frequency-specific and spatially focal modulation of brain activity, their work provides crucial mechanistic insights that are necessary for designing effective tACS protocols to enhance motor control in aging and neurological populations. In a comprehensive review, Koloski et al. (Contribution 9) make a compelling case for using neurophysiological markers, such as cortico-striatal beta oscillations related to reward processing, to inform preclinical TBI research. They argue that these markers are highly translatable between animal models and humans and could be used to develop and test targeted neuromodulation therapies for cognitive impairments, bridging the gap between basic science and clinical application.

## 6. Future Directions and Concluding Remarks

The papers collected in this Special Issue paint a vibrant picture of a field in rapid evolution. Looking ahead, several key research trajectories emerge. The first is the continued push towards closed-loop systems. The ultimate goal is to create integrated systems that can simultaneously record EEG signals, decode user intent or brain state in real time, deliver a therapeutic intervention (e.g., functional electrical stimulation, robotic assistance, or NIBS), and then use subsequent EEG feedback to adapt the intervention on the fly. The work on ML classification and NIBS modulation provides the foundational components for such systems.

A second major direction is the expansion of rehabilitation in naturalistic environments. Technologies such as mobile EEG and VR are critical for moving assessment and therapy out of the constrained laboratory and into settings that more closely mimic the challenges of daily life [2]. Future research should focus on developing robust algorithms that can function amidst the noise and complexity of real-world environments.

Third, the theme of personalization will only grow in importance. As demonstrated by Lacerda et al. and Adolf et al., group-level findings must be refined to account for individual differences in brain injury, neurophysiology, and cognitive profiles. This will require larger datasets, more sophisticated individual-level modeling, and the integration of multimodal data, including genomics, structural imaging, and behavioral assessments [3].

The research presented here collectively demonstrates that EEG is far more than a passive measurement tool; it is an active agent in the rehabilitation process. It provides the essential feedback for BCIs, offers biomarkers to guide treatment selection, and serves as a direct target for neuromodulatory therapies. As we continue to refine our ability to interpret the brain’s complex electrical symphony, EEG will undoubtedly become an even more indispensable component of the neurorehabilitation toolkit, helping us to better understand, restore, and enhance human brain function.
